# A meta-analysis on the prognosis of exosomal miRNAs in all solid tumor patients

**DOI:** 10.1097/MD.0000000000015335

**Published:** 2019-04-19

**Authors:** Jiupeng Zhou, Hui Guo, Yuanli Yang, Yongfeng Zhang, Heng Liu

**Affiliations:** a Xi’an Chest Hospital, Xi’an; b The First Affiliated Hospital of Xi’an Jiaotong University, Shaanxi Province, China.

**Keywords:** exosomal miRNAs, meta-analysis, prognosis, tumor

## Abstract

**Background:**

It has been reported that the encapsulated miRNAs from exosomes are potential biomarkers of tumors prognosis. Yet, the results are controversial, so it is obliged to do a meta-analysis to reach a definite conclusion.

**Materials and methods:**

Studies were searched for published in PubMed, Embase, and Web of Science databases until April 20, 2018. A meta-analysis was conducted to appraise the role of exosomal miRNAs in prognosis of cancer patients.

**Results:**

The different exosomal miRNAs expression was remarkably related to overall survival (OS) (hazard ratio [HR] = 2.02, 95% confidence interval [CI]: 1.84–2.21) and disease-free survival (DFS) (HR = 2.43, 95% CI: 1.86–3.17) of cancer patients. High exosomal miR-21 expression was associated with poor OS (HR = 2.59; 95% CI: 1.71–3.90) and DFS (HR = 1.84; 95% CI: 1.37–2.47). High exosomal miR-451a expression was associated with poor OS (HR = 4.81; 95% CI: 2.33–9.93) and DFS (HR = 2.64; 95% CI: 1.62–4.31). High exosomal miR-1290 expression was associated with poor OS (HR = 1.73; 95% CI: 1.29–2.33). Low exosomal miR-638 expression was associated with poor OS (HR = 2.25; 95% CI: 1.46–3.46).

**Conclusion:**

The expression levels of exosomal miRNAs, particularly miR-21, miR-451a, miR-1290, and miR-638 could strongly predict prognosis of solid tumor patients and might be a potential target for tumor treatment.

## 1 Introduction

MiRNAs are a short (20–24nt) noncoding RNA, which regulate gene expression at the post-transcriptional level in molecular mechanisms by affecting the stability and translation of mRNA. In addition, miRNAs target protein-coding mRNA at the post-transcriptional level by directly dividing mRNA or inhibiting protein synthesis. It has been found that the imbalance of miRNA is related to the occurrence and progression of cancer, implying that miRNAs are possible to be as molecular biomarker for diagnosis and prognosis prediction of cancer.^[1,2]^ Exosomes are nanoscale vesicles (40–100 nm), which originate from the membrane cavity of multiple vesicles and release with cell membrane.^[3]^ As we all know, exosomes contain tissue-specific signals composed of proteins and selective packaged RNA, such as miRNA, which can transfer these components to other cells.^[4]^ The latest evidence have showed that the encapsulated miRNAs from exosomes are potential biomarkers of tumors prognosis, including lung cancer,^[5–7]^ hepatocellular carcinoma,^[8–10]^ hepatoblastoma,^[11,12]^ glioma,^[13]^ colorectal cancer,^[14–20]^ ovarian cancer,^[21]^ prostate cancer,^[22]^ kidney cancer,^[23]^ and pancreatic ductal adenocarcinoma.^[24,25]^ However, the results of some researches were disputable. Some studies demonstrated that the high expression of exosome-delivered miRNAs in human peripheral blood might be associated with the poor prognosis of tumor patients,^[17,22]^ others found that the high expression of exosomal miRNAs was distinctly not related to the unfavorable prognosis of tumor patients.^[6,21]^ Even more, some studies reported that the high expression of exosome-delivered miRNAs might be associated with the favorable prognosis of tumor patients.^[12,16]^ Therefore, the objective of this meta-analysis is to explore the prognosis of exosomal miRNAs in all solid tumor patients and refrain from the possible deviations.

## 2 Materials and methods

Ethical approval was not necessary in meta-analysis.

### 2.1 Literature search strategy

In order to gain the potential qualified research, systematic network document search was aimed at multiple websites database, including Embase, PubMed, and Web of Science until April 20, 2018, and the search keywords were as follows: “exosomal miR”, “exosomes”, “cancer”, “tumor”, “prognosis”, and “survival”. The relevant systematic reviews and references cited in the searched articles were also filted to avoid leaving out any potentially usable researches. Besides, other related articles were also available by examining the reference list by hand.

### 2.2 Selected and removed criteria

The selected criteria were as follows:

1) the expression level of exosomal miRNAs in primary cancerous serum exosomes was measured;
2) dichotomous model was appraised by quantitative polymerase chain reaction (qPCR);
3) proven diagnosis of tumor by histopathology;
4) hazard ratio (HR) and 95% confidence interval (CI) between exosomal miRNAs expression and overall survival (OS) and/or DFS/time to tumor recurrence (TTR) could be picked up directly or calculated indirectly in the study.

The removed criteria were as follows:

1) the letters, experiments, or articles that researched animal models;
2) repeated research publications;
3) the expression of miRNAs was detected in plasma or tumor tissue.

### 2.3 Date extraction and quality evaluation

Two investigators (JP zhou and YL yang) independently extracted the information and data from all eligible studies through cross-check. The data and information were got together from every study using a purpose-designed form: the author, the year of publication, the country, the type of cancer, the overall number of patients, and the standard for high exosomal miRNAs expression. The survival results of both original and adjusted data were OS and/or DFS/TTR. The disagreements between the 2 investigators were settled by means of discussion until an agreement was reached with the third investigators. Quality evaluation was based on the Newcastle–Ottawa quality assessment scale (NOS). The NOS scores varied from 0 to 9. Six points or more were deemed as high quality.

### 2.4 Statistical analysis

The current meta-analysis was carried out using the RevMan5.3 software and Stata SE13.0 software. The prognostic effect of exosomal miRNAs expression was assessed via both adjusted and unadjusted HRs and their 95% CIs of OS and/or DFS/TTR from the primary studies. According to the standard of every study, the expression of exosomal miRNAs was divided into high and low levels. Reported HR and 95% CI were immediately collected from the researches. If HR and 95% CI were not specific in the studies, the means of Tierney et al^[26]^ and Parmar et al^[27]^ were recommended to evaluate HRs and 95% CIs. The heterogeneity among the enrolled studies was performed by I^2^ metric and Q statistic, the *P* value for the Q test <.05 and the I^2^-value >50% were taken into account to be indexes of serious heterogeneity. The random-effects model was selected for the researches with a remarkable heterogeneity (*P* ≤.05, I^2^ ≥50%). Or else, the fixed-effects model was applied (*P* >.05, I^2^ <50%). Publication bias was appraised by Egger test and a funnel plot, and *P* <.05 demonstrated remarkable bias. The *P* value <.05 was deemed statistically significant.

## 3 Results

### 3.1 Studies identification and characteristics of eligible studies

**Figure 1:**
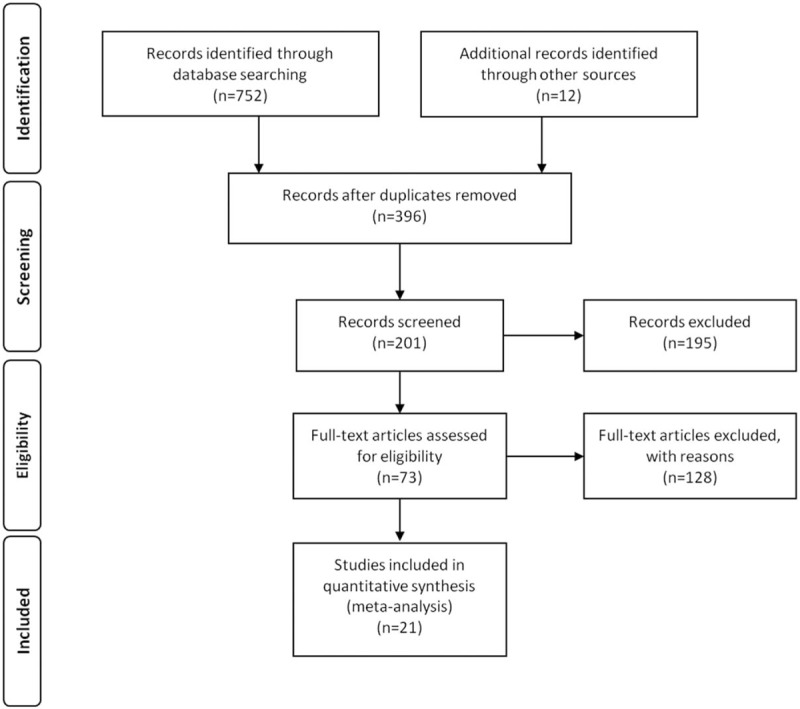
A flowchart presenting the steps of literature retrieval and selection.

After the initial search algorithm, a total of 396 articles were retrieved. Unrelated articles were excluded from headlines and abstracts, and 73 articles were further evaluated in the full text. Those articles, which were review article or case and did not provid survival data to extract, dichotomous variables and valuable data, were excluded. Finally, this meta-analysis contained 21 articles including a total number of 2971 patients (Fig. 1). The average sample number of patients every study was 141.2 (range:30–326). In the 21 studies, 11 of them came from the People's Republic of China, 8 from Japan and 2 from USA In this meta-analysis, 9 different types of cancers were contained, which were 3 lung cancer, 2 hepatoblastoma, 3 hepatocellular carcinoma, 1 glioma, 7 colorectal cancer, 1 ovarian cancer, 1 prostate cancer, 1 kidney cancer, and 2 pancreatic adenocarcinoma respectively (Table 1).

**Table 1:**
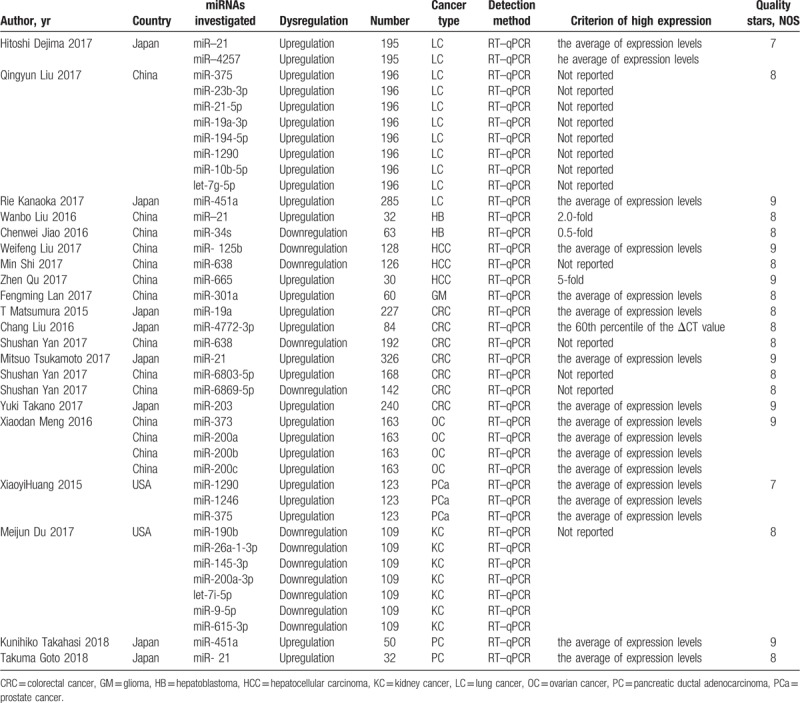
The basic information and data of all included studies in the meta-analysis.

### 3.2 Association between exosomal miRNAs expression and prognosis

**Figure 2:**
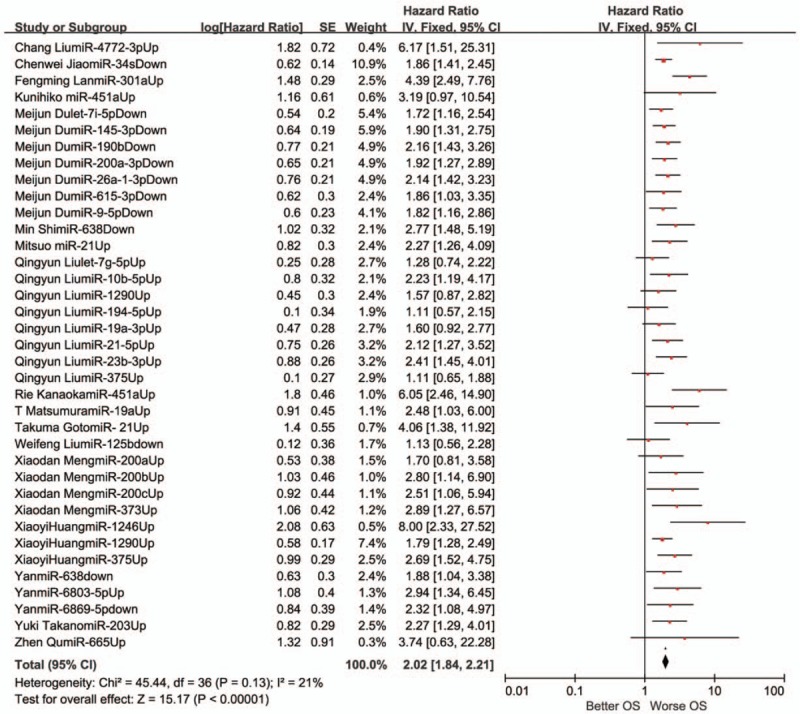
A forest plot for the association between the exosomal miRNA expression levels with OS. OS = overall survival.

**Figure 3:**
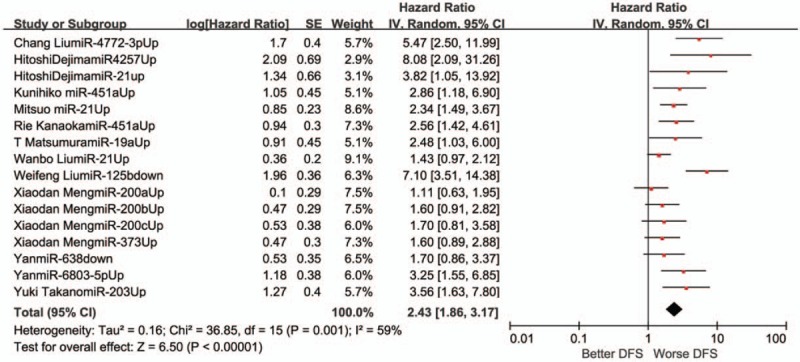
A forest plot for the association between the exosomal miRNA expression levels with DFS. DFS = disease-free survival.

**Figure 4:**
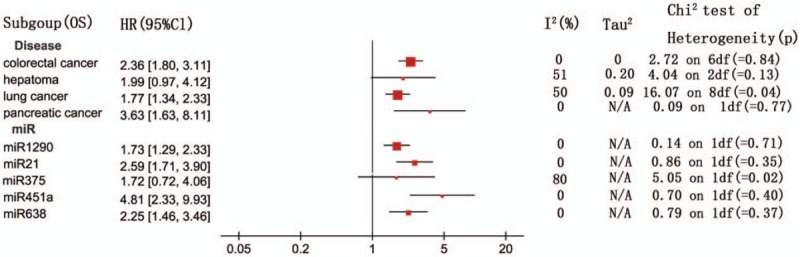
A subgroup analysis for the association between the exosomal miRNA expression levels with OS. OS = overall survival.

**Figure 5:**
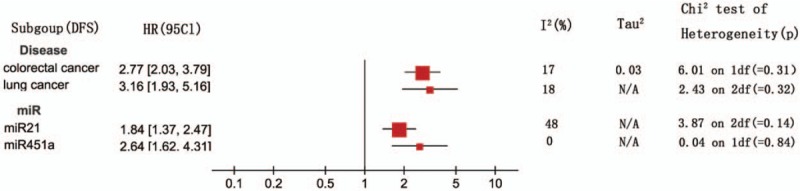
A subgroup analysis for the association between the exosomal miRNA expression levels with DFS. DFS = disease-free survival.

A fixed-effects model was employed to estimate the combined HR with 95% CI for the outcomes of OS in virtue of restricted heterogeneity (I^2^ = 21%, *P* = .13). Pooled HR value (95% CI) of OS associated with different exosomal miRNAs expression was 2.02 (1.84–2.21) in all solid tumor patients (Fig. 2). In addition, the random-effects model was carried out for significant heterogeneity in the studies with DFS (I^2^ = 59%, *P* = .001). The pooled HR value (95% CI) of DFS associated with different exosomal miRNAs expression was 2.43 (1.86–3.17) (Fig. 3) in all solid tumor patients. Poor prognosis was associated with the upregulation of 22 exosomal miRNAs (miR-21, miR-4257, miR-375, miR-23b-3p, miR-21–5p, miR-19a-3p, miR-194–5p, miR-1290, miR-10b-5p, let-7g-5p, miR-451a, miR-665, miR-301a, miR-19a, miR-4772–3p, miR-6803–5p, miR-203, miR-373 miR-200a, miR-200b, miR-200c, miR-1246) and with downregulation of 11 exosomal miRNAs (miR-34 s, miR-125b, miR-638, miR-6869–5p, miR-190b, miR-26a-1–3p, miR-145–3p, miR-200a-3p, let-7i-5p, miR-9–5p, miR-615–3p). Five exosomal miRNAs (miR-21, miR-451a, miR-375, miR-1290 and miR-638) were evaluated by at the least 2 studies. A subgroup meta-analysis was carried out in the relevant studies (Figs. 4 and 5).

The association of exosomal miR-21 with survival outcomes was reported in 4 studies. Two reported OS and 3 reported DFS. The meta-analysis displayed that the high exosomal miR-21 expression was distinctly related to poor OS (a fixed-effect model, HR = 2.59; 95% CI: 1.71–3.90; *P* <.00001; I^2^ = 0%, *P* = .35). The analysis indicated a pooled HR = 1.84 (95% CI: 1.37–2.47, *P* <.00001), demonstrating a poor DFS of high exosomal miR-21 expression (I^2^ = 48%, *P* = .14).

Three studies discussed the relationship between exosomal miR-451a and the result of survival, of which 2 contained OS and 2 contained DFS. The results displayed that the high exosomal miR-451a expression was distinctly related to poor OS (a fixed-effect model, HR = 4.81; 95% CI: 2.33–9.93; *P* <.00001; I^2^ = 0%, *P* = .40). It was demonstrated that the high exosomal miR-451a expression distinctly correlated with poor DFS (a fixed-effect model, HR = 2.64; 95% CI: 1.62–4.31; *P* <.00001; I^2^ = 0%, *P* = .84).

Two studies expounded the part of exosomal miR-1290 in OS of tumor patients. Then, a meta-analysis was conducted on the relation of exosomal miR-1290 expression and OS. This demonstrated that elevatory exosomal miR-1290 expression correlated with poor OS (a fixed-effect model, HR = 1.73; 95% CI: 1.29–2.33; *P* <.001; I^2^ = 0, *P* = .71).

Two studies investigated the association between exosomal miR-375 expression and OS of tumor patients. A meta-analysis was executed on the relationship of exosomal miR-375 expression and OS. The results suggested that abnormal exosomal miR-375 expression was not related to OS (a random-effect model, HR = 1.72; 95% CI: 0.72–4.06; *P* = .23; I^2^ = 80%, *P* = .02).

Two studies included OS for exosomal miR-638 in tumor patients. When a meta-analysis was performed on the relationship of exosomal miR-638 expression and OS, the results indicated that a lower expression of exosomal miR-638 could predict shorter OS (a fixed-effect model, HR = 2.25; 95% CI: 1.46–3.46; *P* <.001; I^2^ = 0, *P* = .37).

### 3.3 Publication bias

**Figure 6:**
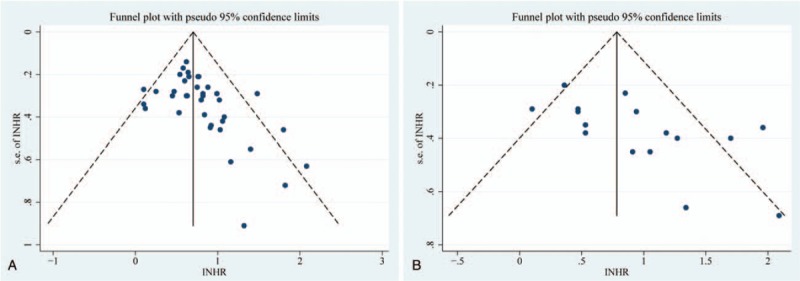
A funnel plot analysis of potential publication bias A: OS; B: DFS. OS = overall survival, DFS = disease-free survival.

Egger test was used to evaluate the publication bias. Egger test demonstrated significant publication bias for OS and DFS in all solid tumor patients (*P* = .004, *P* = .014) (Table 2, Fig. 6). The bias indicated the presence of a language bias, a potential publication bias, an exaggerated estimates by a flawed methodologic design in smaller studies, and a deficiency of publication of small trials with contrary results.

**Table 2:**
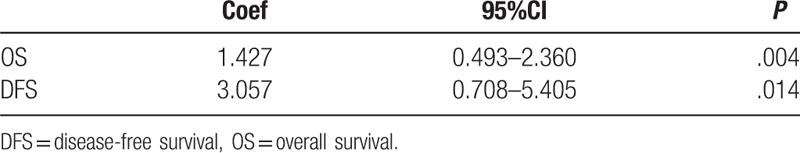
The publication bias test including literatures.

## 4 Discussion and Conclusions

Over the past few years, expression of miRNAs has been assessed in several studies in order to select potential diagnostic and/or prognostic biomarkers in solid tumors. However, the clinical significance of plasma/serum miRNA as a diagnostic biomarker is still disputable.^[28–31]^ The instability of miRNA in plasm/serum may be 1 reason for this controversy. Recent most studies examined the role of exosomal miRNAs in various tumors as a biomarker.

In this study, we systematically analyzed 2971 tumor samples from 21 appropriate articles and reported, for the first time, a group of 33 exosomal miRNAs related to prognosis of all solid tumors. This study aimed to identify the relationship between exosomal miRNAs and solid tumors prognosis, which could be further confirmed in future studies and ultimately assessed before the treatment to improve the treatment of solid tumors.

This research discovered that different exosomal miRNAs expression was associated with OS and DFS in all solid tumor patients, and the pooled HR value (95% CI) was respectivly 2.02 (1.84–2.21) and 2.43 (1.86–3.17). The upregulation of 22 exosomal miRNAs (miR-21, miR–4257, miR-375, miR-23b-3p, miR-21–5p, miR-19a-3p, miR-194–5p, miR-1290, miR-10b-5p, let-7g-5p, miR-451a, miR-665, miR-301a, miR-19a, miR-4772–3p, miR-6803–5p, miR-203, miR-373 miR-200a, miR-200b, miR-200c, miR-1246) and downregulation of 11 exosomal miRNAs (miR-34 s, miR-125b, miR-638, miR-6869–5p, miR-190b, miR-26a-1–3p, miR-145–3p, miR-200a-3p, let-7i-5p, miR-9–5p, miR-615–3p) correlated with poor prognosis.

We conducted the meta-analysis on these 5 exosomal miRNAs (miR-21, miR-451a, miR-375, miR-1290, and miR-638) to determine a pooled conclusion because the 5 exosomal miRNAs were identified by at least 2 studies. The study found that high exosomal miR-21 expression was related to poor OS and DFS. High exosomal miR-451a expression was related to poor OS and DFS. High exosomal miR-375 expression was not related to poor OS. High exosomal miR-1290 expression was related to poor OS. Low exosomal miR-638 expression was related to poor OS.

Aside from the above mentioned exosomal miRNAs, this study also made a systematic study of the relationship between exosomal miRNAs and lung cancer, hepatocellular carcinoma, colorectal cancer, and pancreatic ductal adenocarcinoma prognosis. Figures 4 and 5 summarize the relationship between prognosis of the tumors and exosomal miRNAs.

It is deserved to be mentioned that exosomal miR-21, a well-known miRNA studied in different cancer types. A meta-analysis shows that exosomal miR-21 has a powerful potential to be served as a general biomarker to diagnose cancers.^[32]^ Zhou et al^[33]^ collected 63 published studies and discovered that the increase of miR-21 expression indicated the deterioration of OS in cancers. The mechanism of miR-21 affecting the metastasis and unfavorable prognosis of tumor patients has been reported. MiR-21 can induce tumor by inhibiting the negative regulation of the RAS/MEK/ERK pathway and apoptosis.^[34]^ It is well known that over expression of miR-21 downregulates the expression of PTEN, PDCD4, and TPM1 and promotes cell proliferation and cancer progression.^[34]^ In addition, overexpressed miR-21 enhances the phenotype of cancer stem cells and promotes the invasion, migration, and tumorigenesis in hepatocellular carcinoma.^[35]^ MiR-451a plays a crucial role in the development of human cancers. Kim et al^[36]^ found that miR-451a regulated gliomatous cell proliferation and migration by means of the LKB1-AMPK signaling pathway. Wang et al^[37]^ reported that miR-451a remarkablely inhibited the proliferation of non-small cell lung cancer (NSCLC) cells in vitro, which was partly owing to the downregulation of ras-related protein. Su et al^[38]^ showed that overexpressed miR-451a was related to cell multiplication, migration, and apoptosis in renal cell carcinoma.

Although results of this meta-analysis were supported by powerful proof, some limitations were worth noting. In evaluating the association between exosomal miRNAs and DFS, the heterogeneity detection indicated markedly heterogeneity. The heterogeneity might be caused by different types of tumor and different cut points of high expression of exosomal miRNAs. Moreover, some studies of small sample might also contribute to the formation of heterogeneity. Also, there might be different proportion of advanced tumors in different research centers, and could also be a cause of heterogeneity. In addition, in order to ensure the effectiveness of exosomes, the electron microscopy is usually needed. In the included studies, 6 (5, 7, 8, 10, 17, 25) provided electron microscopic pictures, 3^[6,14,15]^ used electron microscopy without electron microscopic pictures, others^[9,11–13,16,18–24]^ did not show electron microscopy. These contributed to the formation of heterogeneity in evaluating the association between exosomal miRNAs and the survival of tumor.

Second, in Egger test, it also showed that there was a significant *P* value for OS and DFS in all solid tumor patients. This meant that there was publication bias. Prejudice mainly due to the inclination of positive publication and ignore negative results. Publication bias could only increase the unreliability. In addition, the summary of the meeting was excluded, which might lead to publishing bias.

Third, although most data were directly available in research, some studies only offered survival curves, which led to possible deviations between estimated and actual statistical data. In order to reduce the deviation as much as possible, detailed steps had been taken.

At last, population primarily came from East Asia and did not well represent the population all over the world.

Generally speaking, this meta-analysis suggested that several exosomal miRNAs were associated with poor prognosis in all solid tumor patients and served as a promising biomarker to predict survival outcomes, which might be a potential target for tumor treatment. Such molecules should be combined with other clinical and molecular biomarkers to evaluate the optimal treatment option for solid tumor patients. So, larger scale, multicentre and higher quality studies are needed to verify our results.

## Author contributions

Jiupeng Zhou and Yuanli Yang made contributions to conception and design, publication search, quality evaluation, data collection, statistics and manuscript writin; Yongfeng Zhang and Heng Liu made contributions to statistics and editors, and Hui Guo contributed to conception, design, statistics, and editing.

**Conceptualization:** Jiupeng Zhou, Hui Guo.

**Data curation:** Jiupeng Zhou, Hui Guo, Yuanli Yang, Yongfeng Zhang, Heng Liu.

**Software:** Jiupeng Zhou.

**Writing – original draft:** Jiupeng Zhou.

**Writing – review & editing:** Jiupeng Zhou.
